# Phase Response Curve to Light under Ambulatory Conditions: A Pilot Study for Potential Application to Daylight Saving Time Transitions

**DOI:** 10.3390/biology11111584

**Published:** 2022-10-28

**Authors:** Raquel Arguelles-Prieto, Juan Antonio Madrid, Maria Angeles Rol, María Ángeles Bonmatí-Carrión

**Affiliations:** 1 Chronobiology Lab, Department of Physiology, Faculty of Biology, Mare Nostrum Campus, University of Murcia, IUIE, IMIB-Arrixaca, 30100 Murcia, Spain; 2 Ciber Fragilidad y Envejecimiento Saludable (CIBERFES), 28029 Madrid, Spain

**Keywords:** clock change, DST, light exposure, circadian rhythms, ambulatory circadian monitoring, circadian phase, sleep, desynchronization

## Abstract

**Simple Summary:**

Daylight saving time (DST) is adopted for 7 months in different countries around the world. Although the effects of transitions to and from DST have been explored in terms of acute events such as traffic accidents or injuries, the objective consequences of these clock changes, not only on self-selected light exposure patterns but also on how they influence circadian synchronization and sleep, have scarcely been studied. In the present study, we monitored wrist skin temperature, motor activity and light exposure in eight people under free-living conditions during the week before and after both transitions, and applied, for the first time, the phase response curve to light, which also allowed us to infer their effects on sleep patterns. Our results reflect that the transition to DST (which occurs in March in Europe) would be expected to cause a circadian system to advance in order to adapt to the new time; however, synchronizing signals provided by natural and personal light exposure would not favor such an advance. This entails internal desynchronization and longer synchronization times. On the contrary, the transition back to ST (in October in Europe), which implies a phase delay, permits a faster adaptation and maintenance of internal synchronization, despite the fact that exposure to natural light would favor an advance.

**Abstract:**

Several studies have investigated the relationship between daylight saving time (DST) and sleep alterations, psychiatric disorders, cardiovascular events and traffic accidents. However, very few have monitored participants while maintaining their usual lifestyle before and after DST. Considering that DST transitions modify human behavior and, therefore, people’s light exposure patterns, the aim of this study was to investigate the potential effects of DST on circadian variables, considering sleep and, for the first time, the human phase response curve to light. To accomplish this, eight healthy adults (33 ± 11 years old, mean ± SD) were recruited to monitor multivariable circadian markers and light exposure by means of a wearable ambulatory monitoring device: Kronowise^®^. The following night phase markers were calculated: midpoints of the five consecutive hours of maximum wrist temperature (TM5) and the five consecutive hours of minimum time in movement (TL5), sleep onset and offset, as well as sleep duration and light intensity. TM5 for wrist temperature was set as circadian time 0 h, and the balance between advances and delays considering the phase response curve to light was calculated individually before and after both DST transitions. To assess internal desynchronization, the possible shift in TM5 for wrist temperature and TL5 for time in movement were compared. Our results indicate that the transition to DST seems to force the circadian system to produce a phase advance to adapt to the new time. However, the synchronizing signals provided by natural and personal light exposure are not in line with such an advance, which results in internal desynchronization and the need for longer synchronization times. On the contrary, the transition back to ST, which implies a phase delay, is characterized by a faster adaptation and maintenance of internal synchronization, despite the fact that exposure to natural light would favor a phase advance. Considering the pilot nature of this study, further research is needed with higher sample sizes.

## 1. Introduction

Daylight saving time (DST) refers to the practice of advancing clocks one hour during the spring and shifting them back to standard time (ST) during the fall. Germany was the first country to adopt DST in 1916, and it was soon followed by the rest of Europe as World War I went on. Nowadays, approximately 1.6 billion people around the world observe DST [1,2]; they are distributed among forty-nine countries in Europe, seven countries in Asia, five in Australia and the Pacific, two in Africa and two in South America [3]. DST is currently a matter of discussion. In 2018, the European Union conducted a public consultation concerning its degree of acceptance among the citizens, proposing its abolishment and considering the possibility that countries could choose to keep ST or DST throughout the year. Since then, the discussion about DST has extended around the world. However, DST is not merely a political issue, but rather it also has potential effects on the economy, social habits and, more importantly, on health and performance (reviewed in [4]). DST was originally created to save energy by shifting human activity patterns to make better use of daylight, thus reducing the amount of electrical lighting needed during the nighttime [5]. However, this aspect is still controversial, as energy use and human behavioral patterns have changed considerably since DST was first introduced (reviewed in [2,6,7,8,9,10,11,12,13,14,15,16]).

On the other hand, in recent decades, the detrimental effects of DST on health have been investigated [5,17]. In this sense, several studies found a relationship between DST and ST transitions and cardiovascular events, although with moderate effects [18,19,20,21]. The relationship between DST and traffic accidents has also been studied with inconsistent conclusions [22,23,24,25,26,27], and, although few studies address this issue, no effects of DST on psychiatric disorders have been found [28,29], with the exception of an increase in suicides in vulnerable males [30]. However, a few studies have focused on the effects of DST and ST transitions on sleep, finding a resynchronization of waking times and bedtimes after five days [31,32] and after one week [33], with a reduction in the duration and quality of sleep [32,34,35,36,37,38,39], and alterations in sleep onset and offset after the transition to DST [9,40,41,42]. Regarding the shift back to ST, the results are inconclusive as a better adjustment to this transition [42], no change at all in sleep [43,44], compromised sleep (especially for morning chronotypes) [36], beneficial effects on sleep quality [33], and increased sleep duration [37,41] have been reported.

More recently, some studies have used actigraphy to monitor healthy volunteers before and after each transition, highlighting a more significant deterioration of the sleep/wake cycle after the transition to DST, as compared to the transition back to ST [45]. However, when the chronotype is considered, although late types seem to suffer more during the transition to DST, morning types are more sensitive to the transition back to ST in terms of sleep [1,36].

It is widely known that circadian phase changes induced by light depend to a large extent on the timing of exposure. These time-dependent effects can be represented in a phase response curve (PRC) to light, which illustrates the phase shift that light exposure timing evokes in the cyclic period of an oscillation [46]. For humans, this was first established by De Coursey in 1960 [47]. According to this PRC, a phase delay in our sleep or circadian phase markers would occur when light exposure is increased in the afternoon and decreased in the morning with respect to our subjective internal time and vice versa [48]. Several PRCs to light have been published in the literature [46,47,49,50]. One of the most widely accepted was developed by Khalsa et al. (2003) [51].

Taking into account the fact that DST transitions modify human behavior, personal light exposure patterns, and, therefore, the functioning of the circadian system, we aimed to explore the effects of these transitions on circadian synchronization in accordance with the PRC under real-life conditions. We also simultaneously recorded, for the first time, different marker rhythms for which altered synchronization could lead to temporary circadian disruption.

## 2. Materials and Methods

### 2.1. Participants

Eight healthy adults (four females) aged 33 ± 11 years of age (mean ± SD) were recruited to participate in this study. None were taking medication that could influence the results. They were asked to keep their sleep as regular as possible. This study was approved by the University of Murcia Ethics Committee, in line with the standards set by the Declaration of Helsinki. Participants gave written informed consent prior to participation.

### 2.2. Ambulatory Circadian Monitoring

Multivariable ambulatory circadian monitoring (ACM) of wrist skin temperature (WT), motor activity and light exposure, along with sleep parameters, was performed on participants over the course of four days before and one week after the transition to DST and one week before and one week after the shift back to ST. 

A small, watch-like device for ambulatory circadian monitoring (ACM), the “Kronowise 3.0” (Kronohealth SL, Murcia, Spain), was placed on the non-dominant hand in order to reduce masking of circadian variables by motor activity. Wrist skin temperature, triaxial motor acceleration, wrist posture, and light exposure in three spectral bands (visible, blue in the range of 460–490 nm and infrared > 800 nm) were continuously recorded at 10 (acceleration), 1 (skin temperature and light exposure) or 0.033 Hz (1 reading per epoch) for wrist position (for additional details, see Supplementary Information) [52]. 

Participants were instructed to remove the device only for hygienic reasons or to recharge its battery. The recording was split into two parts, coinciding with the onset of DST (at 2:00 am in March and 3:00 am in October, in Europe), and each was analyzed separately. Time was not modified in the recordings before or after the transitions, in order to allow them to be compared. It should be noted that six out of the eight recordings were performed in 2017 and the remaining in 2018. On day 4 and day 5 after the transition from DST, a national holiday occurred in 2017 and 2018, respectively. In addition, in 2018, days 5 and 6 after the transition to DST concurred with the Easter holidays.

### 2.3. Circadian and Sleep Parameters

**Figure 1:**
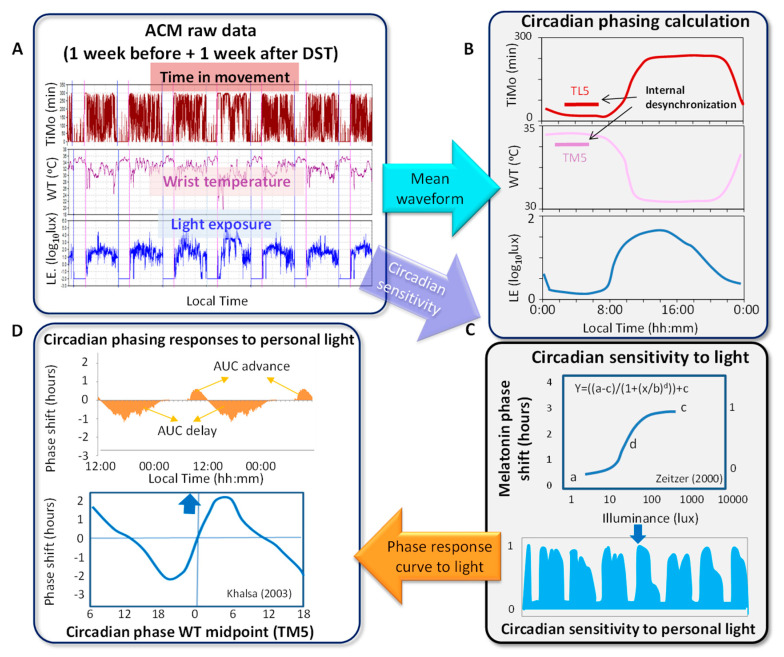
Procedure to analyze light exposure, circadian variables and circadian phasing responses. Once raw data are downloaded from the ACM device (**A**), Kronoware© calculates the daily mean waveforms of wrist temperature (WT), time in movement (TiMo) and exposure to light (LE), as well as their main circadian parameters (**B**). Light exposure is then transformed according to the melatonin phase shift response curve to light intensity (**C**) [58] in order to delimit circadian light sensitivity. The phase response curve to light [51] is then applied to obtain the personalized circadian phasing response to light, which jointly considers both timing and intensity for phase shifting (**D**).

Raw data obtained from ambulatory circadian monitoring (ACM) (Figure 1A) were processed and analyzed with Kronoware 9.0 software (Kronohealth SL, Murcia, Spain) to determine sleep onset, offset and duration. Daily mean waveforms and night phase markers were obtained for wrist skin temperature (WT) (TM5, midpoint of the five consecutive hours with highest values) and for time in movement (TiMo) (TL5, midpoint of the five consecutive hours with lowest values) [53,54,55,56]. The average light exposure pattern (Figure 1B) was also obtained, after eliminating the artifacts, by calculating the daily mean waveform of exposure to light for the week before and the week after each transition (in and from DST). Average sleep parameters (sleep onset, offset and duration) and light exposure were compared before and after each transition. For the purposes of comparison, the local time of the different variables was not changed after the transitions. To compare the individuals’ light exposure to the natural light pattern, daily mean waveforms of exposure to sunlight intensity in Murcia (Spain, 37°59′10″ N 1°07′49″ W) were calculated in March (to DST) and October (back to ST) before and after both transitions (data provided by Solcast API [57]), in parallel with ACM. 

### 2.4. Phase Response Curve

After calculating each daily mean waveform of exposure to light individually, the data were recalculated considering the circadian sensitivity to light estimated by applying the melatonin phase shift response to light intensity [58] (Figure 1C) as follows: 

To calculate the PRCs without losing light exposure information, the daily mean waveform of exposure to light was calculated (by averaging each day) in log lux units. The 4-parameter logistic model of the phase shift curve of plasma melatonin according to light intensity [58] (Figure 1C) was then applied to this mean waveform, in order to delimit the circadian system’s sensitivity to light. Thus, circadian sensitivity was obtained as follows:

(1) $$y=\frac{\left(a-c\right)}{(1\;+\;{\left(x/b\right)}^{d}}+c\;$$

where the parameters estimated by Zeitzer et al. (2000) were:

*a* = 0.240 ± 0.389;

*b* = 120 ± 61.2;

*c* = −3.00 ± 0.235.

Then data were multiplied by the phase response curve to light [51] to obtain the circadian phase response to personal light, according to both intensity and timing (Figure 1D). For that, the PRC [51] was applied to previous light exposure individual data by multiplying each datapoint by the corresponding point on Khalsa’s curve (Figure 1D). Afterward, to estimate the amount of light received in the delay and advance zones, the area under the curve (AUC) of the light received in each part of the PRC was calculated, centering the circadian phase 0 h in the TM5 for WT of each participant. Traditionally, circadian phase 0 h has been established as the time when the minimum core body temperature (CBT) occurs. Since CBT and WT follow opposite patterns [59,60], in our study circadian phase 0 h was established as the TM5 of WT, that is, the central time of the 5 consecutive hours with the highest mean temperature level [61].

The average PRCs were then obtained before and after each transition for the entire group of subjects. To facilitate comparisons before and after time changes, the local time of data recordings was not changed. 

To compare the PRC of personal light exposure with the theoretical response to natural sunlight, Kronowise 3.0 was employed to record sunlight in an artificial light-free area, one week before and one week after each transition (to and from DST). After that, the same process was performed with sunlight before and after each transition, once again centering the circadian phase 0 h of the PRC in the individual TM5 for WT and calculating the corresponding means for each situation.

### 2.5. Chronotype

Chronotype was not considered during recruitment, but its possible influence on the PCR curve was simulated according to the Munich Chronotype Questionnaire (MCTQ [62]). In doing so, zero circadian time was established according to MCTQ scores ranging from 1 to 7, simulating a gradient from morning to evening chronotypes. Next, the AUC in each situation (before and after the two transitions) and the differences between phase advance and phase delay areas were calculated.

### 2.6. Internal Desynchronization

One advantage of using a multivariable monitoring system is the opportunity to assess the degree of synchronization between the different circadian variables. Therefore, the next step was to examine possible internal desynchronization by exploring the daily phase relationship between TiMo and WT [63]. To do this, the night phase marker for TiMo (TL5) was compared to the night phase marker for WT (TM5) for each participant and each day after DST and ST transitions (parameters provided by Kronoware software, Kronohealth, S.L., Murcia, Spain).

### 2.7. Statistical Analysis

Data normality was checked by means of a Shapiro–Wilk test. Student’s *t* tests for related samples were used to compare the TM5 of WT before and after DST transitions and sleep parameters. Repeated measures ANOVA tests, with Bonferroni post-hoc comparisons and light and phase shift as intra-subject factors, were performed to compare changes in phase or light exposure before and after both transitions. Mathematical modeling was used to determine the balance between phase advances and delays according to Munich chronotypes ranging from 1 to 7. TM5 and TL5 for WT and TiMo over the course of the days following DST transitions were compared using a repeated measures ANOVA test, with Bonferroni post-hoc comparisons. TM5 and TL5 were compared within each day using paired T-tests. All statistical tests were performed with SPSS Statistics for Windows, Version 23.0 (IBM Corporation, Armonk, NY, USA).

## 3. Results

### 3.1. Light Exposure and Sleep Patterns

**Figure 2:**
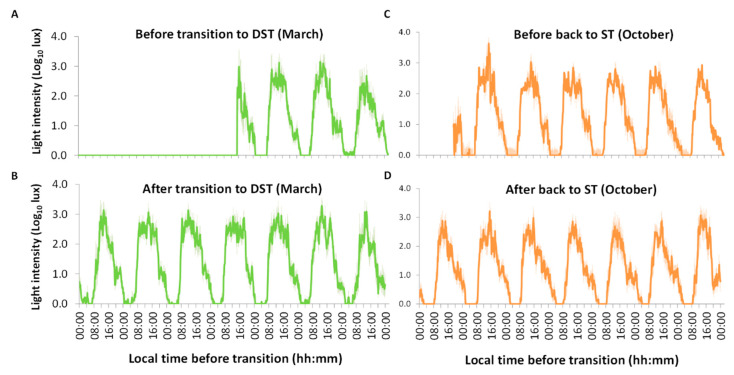
Average (*n* = 8) light exposure in log lux units (± SEM) for all participants (*n* = 8) before (**A**) and after (**B**) transition to DST, and before (**C**) and after (**D**) transition back to ST.

Averaged daily light exposure before and after the transitions is represented in Figure 2. Light exposure shows high values during the day and low levels at night, as expected (Figure 2A). After the transition to DST (Figure 2B), the light remained on for a certain time after bedtime on multiple days. The light exposure pattern for the transition back to ST (Figure 2D) showed more clearly than the transition into DST that participants tended to switch the lights on and off later in comparison with the week before the clock change (Figure 2C).

**Figure 3:**
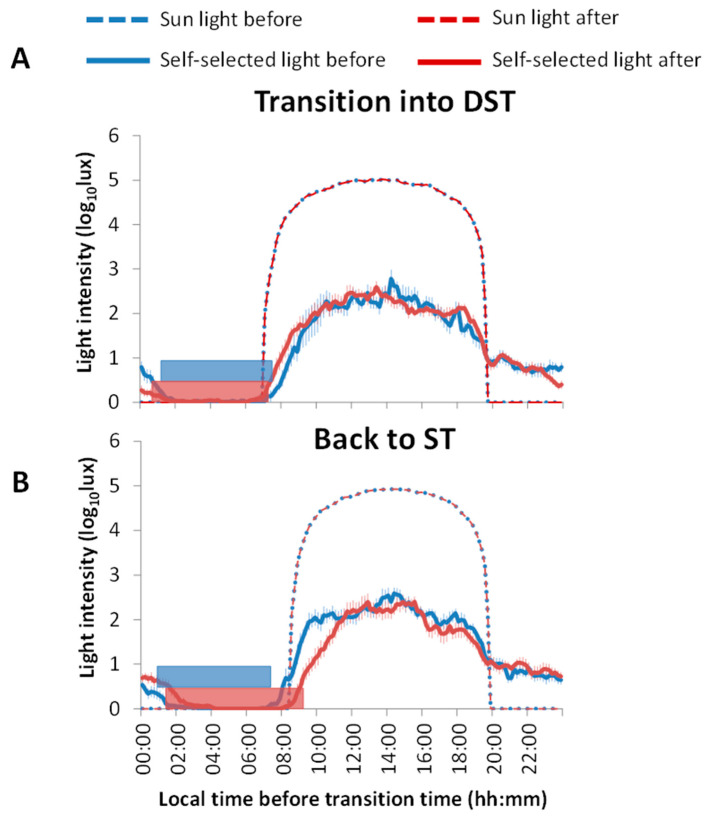
Mean 24-h waveforms of the participants’ (*n* = 8) exposure to artificial light and sunlight (in log lux units) in the same period and location before and after transitions to DST (**A**) and back to ST (**B**). Blue lines indicate the participants’ light exposure before the time change, and red lines show it after the time change. Dotted lines represent the intensity of the sunlight, and horizontal bars show the average sleep period (*n* = 8) before (blue) and after (red) the transitions. Data are expressed as the mean ± SEM.

Figure 3 shows the mean exposure to artificial light and natural sunlight before and after the transitions to DST and back to ST, as well as average sleep duration/timing in all situations (horizontal bars). Sleep onset before and after the transition to DST (Figure 3A) was not significantly different, as was also the case for sleep offset time and sleep duration. In general, participants were exposed to similar light intensities both before and after the transition to DST.

With the transition back to ST (Figure 3B), sleep onset tended to occur later after ST was reestablished (1.51 h ± 0.54 h) as compared to before (0.92 h ± 0.38 h), although the differences were not statistically significant (*p* = 0.1, t = −1.699, DF = 7). This notwithstanding, participants woke up later after the transition back to ST (7.59 h ± 0.38 h before vs. 9.27 h ± 0.32 h after the transition, *p* < 0.005, t = −5.132, DF = 7). Thus, participants slept more after returning to ST (6.67 ± 0.23 h before and 7.76 ± 0.39 h after transition to ST, *p* < 0.05, t = −3.017, DF = 7). After the transition back to ST, the participants tended to be exposed to light later in the morning.

### 3.2. Phase Shifts

**Figure 4:**
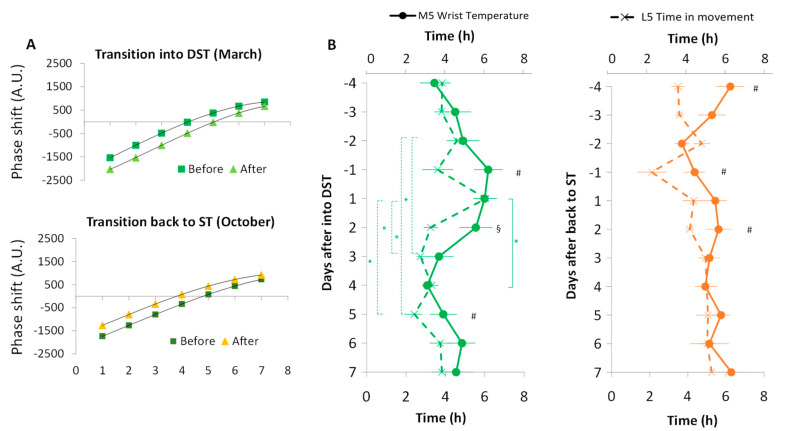
(**A**) Modeling of phase shifts according to the phase response curve [51] (*n* = 8) to natural light before (square) and after (triangle) the transition to DST (**upper** panel) and back to ST (**lower** panel) by chronotype (MCTQ). See the Section 2 for details. (**B**) TM5 for WT (*n* = 8; solid line) and TL5 for TiMo (*n* = 8; dashed line) after transitions to DST (**left** panel) and back to ST (**right** panel). As a reference, the averages of TM5 for WT (circle) and TL5 for TiMo (cross) from the days before the transitions (4 days before DST and 6 days before ST) are included. Data are expressed as the mean ± SEM. * indicates *p* < 0.05 between different days (repeated measures ANOVA, Bonferroni post-hoc comparison). ^#^ indicates *p* < 0.05 between TL5 and TM5 each day (paired *t*-test). ^§^ indicates *p* = 0.05 between TL5 and TM5 each day (paired *t*-test).

Figure 4 shows simulations of the balance between phase advances and delays according to chronotype and natural sunlight exposure when transitioning to and from DST. Before the transition to DST, the balance (0 h shift) between phase advances and delays occurred at approximately 4:00 h, i.e., a chronotype with 4:00 h as zero circadian time. Thus, if morning people were only exposed to sunlight they would be delaying their clock, while evening persons would be advancing their rhythms. After the transition to DST, the zero balance was delayed to 5:00 h, making this clock change even more difficult for morning people (Figure 4A upper panel). However, the transition back to ST (Figure 4A, lower panel) would be easier for morning types since the equilibrium is advanced from almost 5:00 to almost 4:00 h. In order to personalize the phase response curve to light, circadian phase 0 h was set at the individual TM5 for wrist temperature (WT).

To explore the internal desynchronization in the circadian system, two circadian parameters (TL5 and TM5) from two circadian variables with different endogenous components (motor activity (TiMo) and wrist skin temperature, respectively) were compared. Figure 4B shows the evolution of both night phase markers before and after the transition to DST (left) and ST (right). During the four days before the transition to DST, wrist skin temperature TM5 ranged from 3.44 ± 0.74 h to 6.17 ± 0.73 h. Four days after the DST transition, TM5 tended to advance up to 3.08 ± 0.29 h (*p* = 0.013) (Figure 4B, left panel). However, after the transition back to ST (Figure 4B, right panel) no advances or delays were observed between different days.

TL5 also showed an advance during the first five days after the transition to DST, from 6.01 ± 0.58 h on day 1 to 2.41 ± 0.41 h on day 5 (*p* = 0.023) after transition; an advance that was already present and significant on day 3 (2.73 ± 0.20 h, *p* = 0.009). As occurred with TM5, the transition back to ST did not produce any significant phase shift in TL5. In order to explore a possible circadian desynchronization between both rhythms, we considered differences between night phase markers for WT (TM5) and TiMo (TL5). In this sense, it should be noted that day −1 (before transition) yielded significant differences between these phase markers both before the transition to DST and to ST (*p* < 0.01), with TL5 for TiMo occurring earlier than TM5 for WT. With the transition to ST, only day 2 after the transition showed significant differences between both rhythms (TiMo occurring earlier) and these differences disappeared afterward. After the transition to DST, however, TiMo remained advanced with respect to WT at day 5 (*p* < 0.026), showing already a previous tendency on day 2 after the transition (*p* = 0.05).

**Figure 5:**
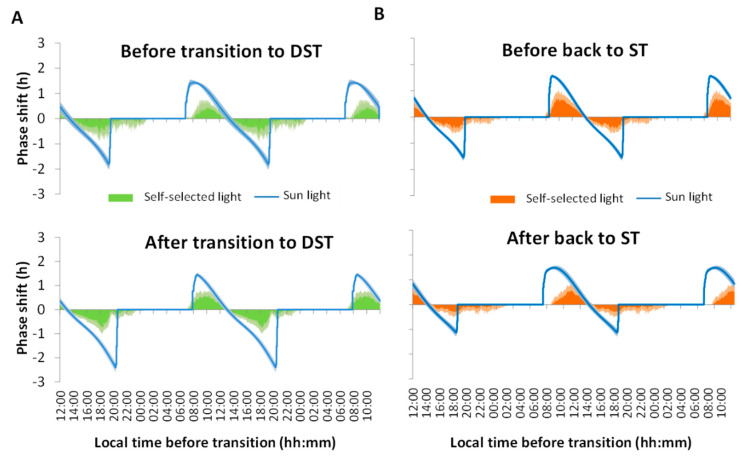
Average theoretical phase advance and phase delay areas under the curve (*n* = 8) before and after DST transition (**A**) and before and after returning to ST (**B**), based on self-selected light exposure (in green) or solar light (in blue) for the corresponding time of the year, centering the circadian phase 0 h on the TM5 for WT of each participant. See the Section 2 for details. Data are expressed as the mean ± SEM.

Regarding the impact of self-selected light exposure considering the PRC to light, before the transition to DST (Figure 5A, upper panel) the participants’ light exposure started at approximately 8:00 h when the sunlight was inducing its maximum phase advance. During the morning, the maximum phase advance achieved by the participants was approximately 0.62 ± 0.13 h, while phase delays during the evening were 0.77 ± 0.15 h. However, after sunset (at approximately 19:30 h) the participants’ exposure to light in the phase delay zone continued until 1:00 h. According to the sunlight PRC (calculated according to previously described formulas and sensitivity curves), assuming no influence of artificial lighting, phase advances would theoretically reach a maximum of 1.43 ± 0.08 h and phase delays of 1.81 ± 0.19 h. On the other hand, after DST (Figure 5A, lower panel), light intensity in the morning produced similar phase advances as before, but in this case, artificial light exposure started one hour earlier (07:00 h) since the sun had not yet risen. Regarding phase delays, the situation was similar to before, with light exposure occurring in the phase delay zone after sunset. Although, in this case, it reached a maximum of almost 2.40 ± 0.18 h and lights were switched off before 00:00 h. 

During the transition back to ST (Figure 5B) and before the clock change (Figure 5B, upper panel), the subjects resumed light exposure at 8:00 h, slightly before sunrise, with a maximum advance of about 0.87 ± 0.14 h and a delay of 0.63 ± 0.09 h. Sunset occurred at approximately 19:30 h, but the participants maintained lights on in the delay zone until 01:00 h, as occurred before the DST transitions. On the contrary, after returning to ST (Figure 5B, lower panel), light exposure started at almost 09:00 h with a maximum advance of approximately 0.66 ± 0.16 h and a maximum delay of about 0.51 ± 0.15 h. According to the sunlight PRC, the sun would rise at 07:30 h and would set at almost 19:00 h, but now the participants’ light exposure lasted until 02:00 h (without changing the local time). 

**Figure 6:**
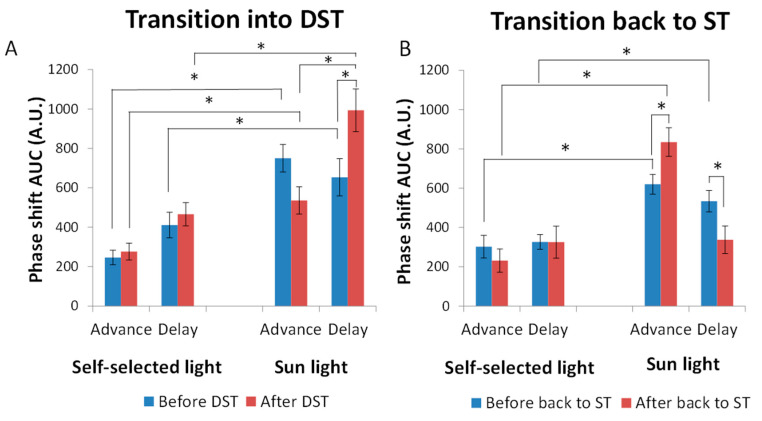
Cumulative average theoretical phase shifts (advances or delays) according to the phase response curve to light [51] (*n* = 8) before (blue) and after (red) the transition to DST (**A**) and after transitioning back to ST (**B**), centering the circadian phase as 0 h on TM5 for WT of each participant (*n* = 8) either for self-selected light or for sunlight. Data are expressed as the mean area under the curve ± SEM. * indicates *p* < 0.05 (repeated measures ANOVA, Bonferroni post-hoc comparisons).

The accumulated amount of light received during the theoretical phase advance or delay zone (calculated according to previously described formulas and sensitivity curves) can be observed more clearly in Figure 6. Overall, during the transition to DST (Figure 6A), the sunlight was greater than the self-selected light exposure and the phase advance area was smaller than the phase delay (*p* < 0.001, F = 46.186, DF = 1 for self-selected light vs. sunlight, and *p* < 0.01, F = 8.748, DF = 1 for advances vs. delays). Before the transition to DST (blue), the exposure in the phase advance zone of the participants’ PRC was lower than that for the sunlight PRC (246 ± 37 A.U. vs. 750 ± 70 A.U., respectively; *p* < 0.001, F = 26.202, DF = 1), but this difference was not so marked for the phase delay (410 ± 64 A.U. vs. 658 ± 94 A.U., respectively; *p* < 0.05, F = 6.287, DF = 1).

After the clock change (Figure 6A, red), differences in phase advances between personal light and sunlight were still noticeable and significant (276 ± 42 A.U. for personal light vs. 653 ± 94 A.U. for sunlight, *p* < 0.05, F = 5.173, DF = 1). However, sunlight almost doubled the phase delay exposure as compared to personal light exposure (993 ± 108 vs. 465 ± 59 A.U., respectively, *p* = 0.001, F = 14.935, DF = 1), and in the case of sunlight, the phase delay exposure was also increased (*p* < 0.05, F = 5.462) and was thus greater (340 A.U.) than the phase advance (*p* < 0.05, F = 10.551, DF = 1). 

Regarding the transition back to ST (Figure 6B), the shifts produced by sunlight cumulative exposure were once again greater than those generated by personal light (*p* < 0.001, F = 37.418, DF = 1), and the phase advances were greater than the delays (*p* < 0.05, F = 6241, DF = 1). The phase delay before the change (blue) was also greater for sunlight than for personal light (533 ± 54 vs. 326 ± 37, *p* < 0.01, F = 8.382, DF = 1), but the differences disappeared after the shift. After ST was reestablished (red), the sunlight generated greater advances than delays (*p* < 0.001, F = 24.376, DF = 1) and the advances increased as compared to before (*p* < 0.05, F = 7.029, DF = 1), while the opposite occurred for delays (*p* < 0.05, F = 5.840, DF = 1).

## 4. Discussion

To our knowledge, this is the first study that applies a human phase response curve to light while participants maintain their usual lifestyle during both transitions to and from DST. Our results show that, in general, the transition to DST has a greater desynchronizing effect on circadian markers than the transition back to ST. 

It has been suggested that adaptation to and from DST is similar to that of a transmeridian flight traveling one time zone to the east or west, respectively [64]; however, the situation is very different. When we travel one hour to the east, our internal time experiences an advance of one hour, and the sun also rises one hour earlier. When transitioning to DST, we need to advance our phase by one hour, although the sun continues to rise at almost the same time as the day before, i.e., we stay where we were instead of exposing our body clock to the new light–dark cycle at our travel destination [4]. This makes resynchronization much more difficult to achieve. Similarly, the change back to ST implies a phase delay of one hour, while the sun continues to rise at approximately the same solar time. 

We must consider that the circadian central pacemaker has a natural tendency to delay its phase [65], so it seems obvious that the transition back to ST would be less disruptive, a priori, and adaptation would be easier than to DST. According to previous studies [31,33] and to our results, this assumption seems to be true, since TM5 for wrist temperature tended to occur later after transitioning back to ST and earlier when changing to DST. Interestingly, although we have not controlled for prior sleep duration, Harrison (2012) [32] pointed out that those participants with prior sleep durations shorter than 7.5 h find it more difficult to fully adjust to ST after DST.

Focusing on the transition to DST, in our study, sleep onset, offset and duration did not change significantly. These results agree with those of other authors [36,40,66] who did not find any significant effect on sleep efficiency following this clock change, although other studies [35,41] found a reduction in sleep duration with the introduction of DST. Michelson (2010) established that the loss of sleep on the Sunday following the transition to DST was less than one hour, as compared to the previous Sunday, while the following Monday had the same average sleep duration as the previous week [37]. This discrepancy could be due to the fact that our participants already slept a reduced number of hours before the transition to DST, as compared to the recommended hours for this age group [67], and so the differences after the transition would be more difficult to observe. On the other hand, after returning to ST, the participants slept more, and their sleep onset and offset occurred later on average than before. These results are along the same lines as those found by Barnes and Wagner (2009) [43], who reported a tendency toward a phase delay and a sleep duration that was 12.4 min longer than on non-phase change days. 

More recently, Tonetti et al. (2013) [45] performed an actigraphic study to explore the effects of the transition to and from DST on the quality of the sleep/wake cycle, finding that the human circadian system adjusts more easily to ST than to DST. It should be noted that previous studies have proven that adaptation to and from DST is chronotype-dependent [34,35,36], with later chronotypes finding it more difficult to adjust to DST, with symptoms including shorter sleep durations [38] and greater diurnal sleepiness [39]. In this sense, the present study has a limitation, since our participants were mainly indefinite and late chronotypes. To counteract this limitation, we performed a simulation of what would occur if participants of different chronotypes (from extremely early to extremely late) were only exposed to sunlight. Our results yielded that transition to DST would be more difficult for morning chronotypes since sunlight would force them to delay their clocks by one hour. The opposite would occur during the shift to ST, where morning chronotypes would have to advance their clocks by one hour. These results do not agree with those reported by Lahti et al. (2006b, 2008) [35,36], probably due to the potential influence of artificial lighting which has not been included in these simulations. In fact, and according to daily mean waveforms of exposure to light (and considering that timing was not changed after transitions to facilitate comparisons), the levels of light intensity were similar but lasted several hours after sunset, reflecting the influence of the use of artificial lighting. 

Therefore, our results bring to light the importance of considering the influence of artificial lighting, since there were large differences in the phase shifts generated by self-selected light or sunlight [42]. Before the transition to DST, sunlight in the mornings occurs progressively earlier and sunset progressively later, according to the official time [36,48], but exposure in the phase advance zone is greater than that for the delays. However, this is contradictory to personal habits, since exposure to light in the evening is greater than in the morning, both before and after the change. After the transition, natural light in the afternoon increases since the sun sets later in relation to the official time, favoring phase delays.

Before returning to ST, the light received during periods of phase advance and delay was almost balanced, and the re-establishment of ST involves receiving more natural light in the mornings than in the evenings since the sun sets earlier. However, again, this fact was not parallel to personal light exposure which remained almost balanced between exposures in delay and advance zones as a consequence of using artificial lighting in the evening to counteract the absence of natural light. It is interesting to note that this same balance also supports the adoption of standard time on a permanent basis, as opposed to DST.

Regarding internal synchronization, our results also support that adaptation to ST change is easier than to DST. Although it should be noted that there was a misalignment the day before both transitions, in the latter case, desynchronization between motor activity and wrist skin temperature seems to appear up to five days after the transition. On the day of the clock change, both rhythms suffered a delay, although they seemed to resynchronize during the first workdays after the change, advancing their phase progressively. However, when the weekend arrived, participants tended to return to the internal timing shown before the transition. This seems to reflect that volunteers forced themselves to wake up earlier due to their work schedule, but their internal time, in fact, was not synchronized with the new official timing. Differences were greater in the midpoint of the five hours of lower activity (TL5) for time in movement, since there was also a misalignment with the values from the previous week and day 5 after transition, probably because this variable is more dependent on willingness and then more affected by clock change. However, differences in TM5 for wrist temperature were smaller, probably because temperature possesses a more endogenous component. On the other hand, during the transition back to ST, both phase markers (time in movement and wrist temperature) remained close to each other, supporting that this transition is less disruptive, and adaptation is easier as compared to DST. A more rapid adjustment is also reached by the variable (TiMo) with a less endogenous component [1,45,48]. In any case, both transitions to DST and back to ST have effects on circadian variables, corroborating that, in terms of circadian synchronization, DST should be abolished in favor of permanent ST since it matches the sun time more accurately and, therefore, it is more respectful of our internal rhythms [4,68,69,70,71].

This pilot study has some limitations. First, although identified, we did not control for factors such as non-working days, chronotypes and social jet lag. It should be noted, however, that in Spain, the transition back to ST (the last Sunday of October) is usually close to a holiday (1 November), which could have affected the results by increasing time spent in bed [72] due to the lack of work duties. In addition, we used a phase response curve obtained under laboratory conditions and in response to light pulses of a limited number of hours to determine the synchronizing effects of continuous light exposure on subjects under natural life conditions. In any event, these results reflect that, despite individual differences, one week after the transition to DST adaptation was not completed, while adaptation back to ST was faster, which is in accordance with previous studies [73]. Finally, this pilot study is based on a small sample. Further and larger studies are needed to confirm our results.

## 5. Conclusions

As far as we know, this is the first study that applies a phase response curve to light in participants whose light exposure has been registered under normal living conditions during the transition to and from DST. Transition to DST increases light which could generate delays, both for self-selected and natural light, which may eventually produce desynchronization between motor activity and wrist temperature rhythms. Similarly, returning to ST, phase advances are favored according to the available sunlight, while self-selected light exposure remains balanced. Furthermore, after returning to ST, sleep duration is increased and the internal desynchronization, seen as the differences in timing for wrist temperature and time in movement, does not seem to be as great as for the DST transition, making the adaptation easier.

Considering that both transitions have effects on our circadian rhythms, our results support abolishing DST in favor of permanent ST and reflect the importance of considering the individual use of artificial lighting when assessing the effects of DST/ST transitions on the circadian system. We would also highlight the importance of using multivariable recordings that make it possible to evaluate internal synchronization.

## 6. Patents

Kronowise 3.0 corresponds to the commercial version of the patent P201031894 (BOPI 22/3/2013) held by the University of Murcia and licensed to Kronohealth, S.L.

## Data Availability

Not applicable.

## References

[1] Kantermann T., Juda M., Merrow M., Roenneberg T. (2007). The Human Circadian Clock’s Seasonal Adjustment Is Disrupted by Daylight Saving Time. Curr. Biol..

[2] Aries M.B.C., Newsham G.R. (2008). Effect of daylight saving time on lighting energy use: A literature review. Energy Policy.

[3] Thorsen S., Bikos K., Brastad I., Buckle A., Gundersen M., Jones G., Kher A., Rehberger G. Time and Date-Daylight Saving Time. https://www.timeanddate.com/time/dst/.

[4] Roenneberg T., Winnebeck E.C., Klerman E.B. (2019). Daylight saving time and artificial time zones—A battle between biological and social times. Front. Physiol..

[5] Gaski J.F., Sagarin J. (2011). Detrimental effects of daylight-saving time on SAT scores. J. Neurosci. Psychol. Econ..

[6] Kotchen M.J., Grant L.E. (2008). Does Daylight Saving Time Save Energy? Evidence from a Natural Experiment in Indiana. Natl. Bur. Econ. Res..

[7] Choi S., Pellen A., Masson V. (2017). How does daylight saving time affect electricity demand? An answer using aggregate data from a natural experiment in Western Australia. Energy Econ..

[8] Karasu S. (2010). The effect of daylight saving time options on electricity consumption of Turkey. Energy.

[9] Alencar J.C.N., Leocadio-Miguel M.A., Duarte L.L., Louzada F., Fontenele Araujo J., Pedrazzoli M. (2017). Self-reported discomfort associated with Daylight Saving Time in Brazilian tropical and subtropical zones. Ann. Hum. Biol..

[10] Bellia L., Acosta I., Campano M.Á., Fragliasso F. (2020). Impact of daylight saving time on lighting energy consumption and on the biological clock for occupants in office buildings. Sol. Energy.

[11] Küfeoğlu S., Üçler Ş., Eskicioğlu F., Öztürk E.B., Chen H. (2021). Daylight Saving Time policy and energy consumption. Energy Rep..

[12] Hill S.I., Desobry F., Garnsey E.W., Chong Y.F. (2010). The impact on energy consumption of daylight saving clock changes. Energy Policy.

[13] Verdejo H., Becker C., Echiburu D., Escudero W., Fucks E., Jose Reveco M. (2016). Impact of daylight saving time on the Chilean residential consumption. Energy Policy.

[14] Kudela P., Havranek T., Herman D., Irsova Z. (2020). Does daylight saving time save electricity? Evidence from Slovakia. Energy Policy.

[15] Awad Momani M., Yatim B., Ali M.A.M. (2009). The impact of the daylight saving time on electricity consumption—A case study from Jordan. Energy Policy.

[16] Mirza F.M., Bergland O. (2011). The impact of daylight saving time on electricity consumption: Evidence from southern Norway and Sweden. Energy Policy.

[17] Harrison Y. (2013). The impact of daylight saving time on sleep and related behaviours. Sleep Med. Rev..

[18] Tarquini R., Carbone A., Martinez M., Mazzoccoli G. (2019). Daylight saving time and circadian rhythms in the neuro-endocrine-immune system: Impact on cardiovascular health. Intern. Emerg. Med..

[19] Manfredini R., Fabbian F., Cappadona R., Modesti P.A. (2018). Daylight saving time, circadian rhythms, and cardiovascular health. Intern. Emerg. Med..

[20] Manfredini R., Fabbian F., Cappadona R., De Giorgi A., Bravi F., Carradori T., Flacco M.E., Manzoli L. (2019). Daylight Saving Time and acute myocardial infarction: A meta-analysis. J. Clin. Med..

[21] Sipilä J.O.T., Ruuskanen J.O., Rautava P., Kytö V. (2016). Changes in ischemic stroke occurrence following daylight saving time transitions. Sleep Med..

[22] Varughese J., Allen R.P. (2001). Fatal accidents following changes in daylight savings time: The American experience. Sleep Med..

[23] Coren S. (1996). Daylight savings time and traffic accidents. N. Engl. J. Med..

[24] Ferguson S.A., Preusser D.F., Lund A.K., Zador P.L., Ulmer R.G. (1995). Daylight Saving Time and Motor Vehicle Crashes: The Reduction in Pedestrian and Vehicle Occupant Fatalities. Am. J. Public Health.

[25] Lambe M., Cummings P. (2000). The shift to and from daylight savings time and motor vehicle crashes. Accid. Anal. Prev..

[26] Huang A., Levinson D. (2010). The effects of daylight saving time on vehicle crashes in Minnesota. J. Saf. Res..

[27] Prats-Uribe A., Tobías A., Prieto-Alhambra D. (2018). Excess Risk of Fatal Road Traffic Accidents on the Day of Daylight Saving Time Change. Epidemiology.

[28] Lahti T.A., Haukka J., Lönnqvist J., Partonen T. (2008). Daylight saving time transitions and hospital treatments due to accidents or manic episodes. BMC Public Health.

[29] Shapiro C.M., Blake F., Fossey E., Adams B. (1990). Daylight saving time in psychiatric illness. J. Affect. Disord..

[30] Berk M., Dodd S., Hallam K., Berk L., Gleeson J., Henry M. (2008). Small shifts in diurnal rhythms are associated with an increase in suicide: The effect of daylight saving. Sleep Biol. Rhythm..

[31] Monk T.H., Folkard S. (1976). Adjusting to the changes to and from Daylight Saving Time. Nature.

[32] Harrison Y. (2012). Individual response to the end of Daylight Saving Time is largely dependent on habitual sleep duration. Biol. Rhythm Res..

[33] Monk T.H., Aplin L.C. (1980). Spring and Autumn daylight saving time changes: Studies of adjustment in sleep timings, mood, and efficiency. Ergonomics.

[34] Lahti T.A., Leppämäki S., Lönnqvist J., Partonen T. (2006). Transition to daylight saving time reduces sleep duration plus sleep efficiency of the deprived sleep. Neurosci. Lett..

[35] Lahti T.A., Leppämäki S., Ojanen S.M., Haukka J., Tuulio-Henriksson A., Lönnqvist J., Partonen T. (2006). Transition into daylight saving time influences the fragmentation of the rest-activity cycle. J. Circadian Rhythm..

[36] Lahti T.A., Leppämäki S., Lönnqvist J., Partonen T. (2008). Transitions into and out of daylight saving time compromise sleep and the rest-activity cycles. BMC Physiol..

[37] Michelson W. (2010). Sleep Time: Media Hype vs. Diary Data. Soc. Indic. Res..

[38] Allebrandt K.V., Teder-Laving M., Kantermann T., Peters A., Campbell H., Rudan I., Wilson J.F., Metspalu A., Roenneberg T. (2014). Chronotype and sleep duration: The influence of season of assessment. Chronobiol. Int..

[39] Schneider A.M., Randler C. (2009). Daytime sleepiness during transition into daylight saving time in adolescents: Are owls higher at risk?. Sleep Med..

[40] Toth Quintilham M.C., Adamowicz T., Pereira É.F., Pedrazzoli M., Louzada F.M. (2014). Does the transition into daylight saving time really cause partial sleep deprivation?. Ann. Hum. Biol..

[41] Johnsen M.T., Wynn R., Allebrandt K., Bratlid T. (2013). Lack of major seasonal variations in self reported sleep-wake rhythms and chronotypes among middle aged and older people at 69 degrees North: The Tromsø Study. Sleep Med..

[42] Shochat T., Santhi N., Herer P., Flavell S.A., Skeldon A.C., Dijk D.J. (2019). Sleep timing in late autumn and late spring associates with light exposure rather than Sun time in college students. Front. Neurosci..

[43] Barnes C.M., Wagner D.T. (2009). Changing to Daylight Saving Time cuts into sleep and increases workplace injuries. J. Appl. Psychol..

[44] Lo J.C., Leong R.L.F., Loh K.K., Dijk D.J., Chee M.W.L. (2014). Young adults’ sleep duration on work days: Differences between East and West. Front. Neurol..

[45] Tonetti L., Erbacci A., Fabbri M., Martoni M., Natale V. (2013). Effects of transitions into and out of daylight saving time on the quality of the sleep/wake cycle: An actigraphic study in healthy university students. Chronobiol. Int..

[46] Minors D.S., Waterhourse J.M., Wirz-Justice A. (1991). A human phase-response curve to light. Neurosci. Lett..

[47] De Coursey P.J. (1960). Daily Light Sensitivity Rhythm in a Rodent. Science.

[48] Meira e Cruz M., Miyazawa M., Manfredini R., Cardinali D., Madrid J.A., Reiter R., Araujo J.F., Agostinho R., Acuña-Castroviejo D. (2019). Impact of Daylight Saving Time on circadian timing system: An expert statement. Eur. J. Intern. Med..

[49] Blattner M.S., Mahoney M.M. (2013). Photic phase-response curve in 2 strains of mice with impaired responsiveness to estrogens. J. Biol. Rhythm..

[50] Revell V.L., Molina T.A., Eastman C.I. (2012). Human phase response curve to intermittent blue light using a commercially available device. J. Physiol..

[51] Khalsa S.B.S., Jewett M.E., Cajochen C., Czeisler C.A. (2003). A phase response curve to single bright light pulses in human subjects. J. Physiol..

[52] Madrid-Navarro C.J., Escamilla-Sevilla F., Mínguez-Castellanos A., Campos M., Ruiz-Abellán F., Madrid J.A., Rol M.A. (2018). Multidimensional circadian monitoring by wearable biosensors in Parkinson’s disease. Front. Neurol..

[53] Ortiz-Tudela E., Martinez-Nicolas A., Campos M., Rol M.Á., Madrid J.A. (2010). A new integrated variable based on thermometry, actimetry and body position (TAP) to evaluate circadian system status in humans. PLoS Comput. Biol..

[54] Bonmati-Carrion M.A., Middleton B., Revell V.L., Skene D.J., Rol M.A., Madrid J.A. (2015). Validation of an innovative method, based on tilt sensing, for the assessment of activity and body position. Chronobiol. Int..

[55] Bonmati-Carrion M.A., Middleton B., Revell V., Skene D.J., Rol M.A., Madrid J.A. (2014). Circadian phase assessment by ambulatory monitoring in humans: Correlation with dim light melatonin onset. Chronobiol. Int..

[56] Bonmati-Carrion M.-A., Revell V.L., Cook T., Welch T., Rol M.Á., Skene D.J., Madrid J.A. (2020). Living without temporal cues: A case study. Front. Physiol..

[57] Solar Forecasting & Solar Irradiance Data. https://solcast.com/.

[58] Zeitzer J.M., Dijk D.J., Kronauer R.E., Brown E.N., Czeisler C.A. (2000). Sensitivity of the human circadian pacemaker to nocturnal light: Melatonin phase resetting and suppression. J. Physiol..

[59] Sarabia J.A., Rol M.Á., Mendiola P., Madrid J.A. (2008). Circadian rhythm of wrist temperature in normal-living subjects A candidate of new index of the circadian system. Physiol. Behav..

[60] Krauchi K., Deboer T. (2010). The interrelationship between sleep regulation and thermoregulation. Front. Biosci.-Landmark Ed..

[61] Witting W., Kwa I.H., Eikelenboom P., Mirmiran M., Swaab D.F. (1990). Alteration in the circadian rest-activity rhythm in aging and alzheimer’s disease. Biol. Psychiatry.

[62] Roenneberg T., Wirz-Justice A., Merrow M. (2003). Life between clocks: Daily temporal patterns of human chronotypes. J. Biol. Rhythm..

[63] Bonmati-Carrion M.A., Hild K., Isherwood C., Sweeney S.J., Revell V.L., Skene D.J., Rol M.A., Madrid J.A. (2016). Relationship between human pupillary light reflex and circadian system status. PLoS ONE.

[64] Klein K.E.E., Wegmann H.M.M., Hunt B.I.I. (1972). Desynchronization of body temperature and performance circadian rhythm as a result of outgoing and homegoing transmeridian flights. Aerosp. Med..

[65] Czeisler C.A., Duffy J.F., Shanahan T.L., Brown E.N., Mitchell J.F., Rimmer D.W., Ronda J.M., Silva E.J., Allan J.S., Emens J.S. (1999). Stability, precision, and near-24-hour period of the human circadian pacemaker. Science.

[66] Nicholson A.N., Stone B.M. (1978). Adaptation of sleep to British Summer Time [proceedings]. J. Physiol..

[67] Watson N.F., Badr M.S., Belenky G., Bliwise D.L., Buxton O.M., Buysse D., Dinges D.F., Gangwisch J., Grandner M.A., Kushida C. (2015). Recommended amount of sleep for a healthy adult: A joint consensus statement of the American Academy of Sleep Medicine and Sleep Research Society. Sleep.

[68] Sládek M., Kudrnáčová Röschová M., Adámková V., Hamplová D., Sumová A. (2020). Chronotype assessment via a large scale socio-demographic survey favours yearlong Standard time over Daylight Saving Time in central Europe. Sci. Rep..

[69] Poteser M., Moshammer H. (2020). Daylight saving time transitions: Impact on total mortality. Int. J. Environ. Res. Public Health.

[70] McDougal D.H., Gamlin P.D. (2010). The influence of intrinsically-photosensitive retinal ganglion cells on the spectral sensitivity and response dynamics of the human pupillary light reflex. Vis. Res..

[71] Danilenko K.V., Kobelev E., Semenova E.A., Aftanas L.I. (2019). Summer-winter difference in 24-h melatonin rhythms in subjects on a 5-workdays schedule in Siberia without daylight saving time transitions. Physiol. Behav..

[72] Bonmatí-Carrión M.-Á., Casado-Ramírez E., Moreno-Casbas M.T., Campos M., Consortium M., Madrid J.A., Rol M.-A. (2022). Living at the wrong time: Effects of unmatching official time in Portugal and Western Spain. Biology.

[73] Valdez P., Ramírez C., García A. (2003). Adjustment of the sleep-wake cycle to small (1–2 h) changes in schedule. Biol. Rhythm Res..

